# Is Arthropod Saliva the Achilles’ Heel of Vector-Borne Diseases?

**DOI:** 10.3389/fimmu.2013.00255

**Published:** 2013-08-30

**Authors:** Wolfgang W. Leitner, Tonu Wali, Adriana Costero-Saint Denis

**Affiliations:** ^1^Division on Allergy, Immunology and Transplantation, National Institute of Allergy and Infectious Diseases, National Institutes of Health, Bethesda, MD, USA; ^2^Division of Microbiology and Infectious Diseases, National Institute of Allergy and Infectious Diseases, National Institutes of Health, Bethesda, MD, USA

**Keywords:** vector-borne disease, vaccine, arthropod saliva, immunomodulation, malaria, leishmaniasis, dengue, Lyme disease

Vector-borne pathogens are deposited into the skin of the vertebrate host animal during the process of blood feeding. The pathogen establishes an infection from this site rather than by having direct access to the host’s circulatory system. In an attempt to simplify the complex interactions between multiple species (vertebrate host, arthropod vector, and microbial pathogen) that occur during the blood meal and following the deposition of pathogen in the host, researchers routinely use artificial animal models, which do not account for a number of potential parameters. The delivery of isolated, purified pathogens by injection rather than natural infection by pathogen-carrying vectors introduces significant artifacts (1, 2). Therefore, insights gained from such models are somewhat limited and, not surprisingly, successes with experimental vaccines developed by using those models have been very difficult to replicate in field trials. In addition to the route of delivery, the immune and disease-status of the vaccinee (e.g., chronic infection with parasitic organisms), the heterogeneity of the pathogen (i.e., exposure to different pathogen strains in the field compared to the challenge with a defined pathogen strain in the laboratory), an important difference between vector-borne pathogens delivered by needle and syringe after their isolation from an infected vector (artificial) and by an arthropod vector (natural) is the presence of arthropod saliva in the latter scenario. The small amounts of vector-derived molecules in the infectious inoculum can significantly change the infectivity of the vector-borne pathogen as first described for *Leishmania* more than two decades ago (3). Saliva molecules delivered to the bite site together with a vector-borne pathogen have been shown to modulate or derail vertebrate immune responses resulting in a local microenvironment that favors the establishment of a vector-borne disease. For example, tick-derived saliva factors appear to inhibit inflammatory cytokine secretion thus preventing efficient immune responses against tick-borne *Rickettsia* (4); a defined molecule in the saliva of the *Aedes aegypti* mosquito (SAAG-4) has the potential to alter the Th-profile of the bite-induced immune response likely rendering the host unable to effectively eliminate vector-borne viruses (5); and sand fly saliva has a caspase-dependent, pro-apoptotic effect on neutrophils resulting in an infection of the host with increased numbers of *Leishmania* parasites (6). Vector saliva can even exhibit its effect on the course of an infection when delivered separately from the infectious inoculum, as demonstrated by the injection of purified *Plasmodium* parasites followed by the bite of a non-infected mosquito and thus the delivery of salivary proteins in “trans” (7). Finally, even a temporal separation of saliva- and pathogen-delivery cannot eliminate effects of arthropod saliva on a subsequent infection with a vector-borne disease (8).

Numerous reports have documented the potent and pleiotropic effects of the saliva of blood-feeding arthropods, which include anti-coagulation, vasodilation anti-inflammation [reviewed by (9)], calling into question how minute amounts of proteins in the inoculum that is delivered during a blood meal could significantly alter the host’s immune response against the vector-delivered pathogen. This observation is particularly puzzling considering that vector saliva primarily evolved to assist the arthropod in obtaining a blood meal and not to facilitate the infection of the vertebrate host with a vector-borne pathogen. The main effect of immunomodulatory saliva components in regard to infection appears to be temporary and local, altering immune responses at the bite site in the skin long enough to allow the vector to feed and for small numbers of pathogenic organisms to establish an infection. In addition to these indirect effects, certain salivary proteins, such as Salp15 in ticks, can be used by the pathogen (*Borrelia burgdorferi* in this case) to directly protect it from antibody-mediated killing when the pathogen coats itself with the vector-derived protein (10). These findings explain why only a small number of *Plasmodium* parasites injected by a mosquito and in the presence of arthropod saliva causes malaria infection, but large numbers of isolated (saliva-free) sporozoites have to be injected by needle and syringe to accomplish the same task. This is not only the case when injecting the parasites intravenously but also when infecting the host through the same route as the mosquito, the skin (11). Similar findings were made with *Leishmania* parasites, which efficiently establish infections after injection only when co-delivered with sand fly saliva (12). Observations such as these have prompted investigators to consider saliva proteins as vaccine candidates based on the hypothesis that neutralizing these immunomodulatory molecules might eliminate their ability to provide an immunological cloak and allow innate immune responses in the vertebrate skin to successfully eliminate the small infectious inoculum. The idea is further supported by numerous reports going back several decades that the pre-exposure to saliva from certain vectors can induce protection against subsequent infectious bites. Using vector saliva rather than pathogen-derived antigens as vaccine candidates has a number of attractive advantages, including: (1) protective immunity might be independent of the pathogen strain; (2) vaccine efficacy might not be abrogated by escape mutants, i.e., vector-delivered viruses, bacteria, or parasites with mutations in the vaccine-encoded antigen; and (3) the efficacy of some of these vaccines would not be limited to the vertebrate host, but may extend to the vector itself. Examples for the latter point are the tick mannose-binding lectin TSLPI, which assists *Borrelia* (the etiologic agent of Lyme disease) in establishing infection in the vertebrate host, and is also crucial for vector colonization (13), as well as the tick protein Salp25D, which is essential for the acquisition of *Borrelia* by the vector and which can be neutralized by the blood meal of an immunized host (14). Thus, vaccines targeting such molecules might act both as vaccines that prevent infection in the vertebrate host and as transmission-blocking vaccines which mediate their effects in the vector and preclude transmission.

A number of reports describe the potential of vaccines based on salivary antigens (15–17), and with the proof-of-concept for saliva antigen-based vaccines well established, the question is: why has this research area not advanced more rapidly and produced more vaccines for the long list of vector-borne diseases? Despite the successful identification and classification of vast numbers of salivary proteins in many different vectors, the list of saliva proteins with defined immunological functions which could serve as candidates for rationally designed vaccines is very short. The matter is further complicated by studies reporting contradictory effects of salivary proteins, in part because some studies use recombinant or isolated saliva proteins while crude salivary gland extracts are used in others. For example, while one study was unable to detect an effect of mosquito saliva on malaria infection (18), other investigators, using the same parasite species, report drastic effects of mosquito saliva on the severity of the infection (7). Some of the reasons for the observed differences between experimental systems were discussed at a workshop in 2012 (19), resulting in strong recommendations to standardize these model systems but also to focus on the analysis of individual saliva components rather than heterogenous and insufficiently characterized salivary gland extracts.

It is also important to consider that immune responses to some salivary antigens may not be beneficial for the host and thus certain salivary proteins would not be suitable as vaccine candidates. For example, immunization with the mosquito salivary protein D7 results in enhanced pathogenesis from mosquito-transmitted West Nile Virus infection (20). The authors of this study concluded that the selection of salivary protein vaccine candidates on the basis of abundance and immunogenicity does not predict efficacy and that such findings emphasize the need to better understand the function of individual salivary gland antigens before they can be considered as vaccine candidates. Furthermore, some vector saliva proteins exhibit potent immunosuppressive activity [reviewed by (21)], which may interfere with the induction of vaccine-induced immune responses to these proteins. This may require the modification of salivary protein which are used as a vaccine candidates in order to inactivate the molecule’s biological function, a relatively straight-forward approach already employed for toxins which are used as vaccines (22).

How do immune responses against vector saliva proteins, induced by natural exposure or vaccines, protect the mammalian host against infection by vector-borne pathogens? The simplest explanation is antibody-mediated inhibition and neutralization of those saliva proteins which either facilitate blood feeding (indirect effect on vector transmission) or suppress immune responses at the bite site (direct effect on vector-borne pathogen). However, at least in the case of *Leishmania*, anti-saliva antibodies did not correlate with protection of rodents (23). Instead, a vaccine-primed, rapid Th1-polarized cellular response at the bite site appeared to establish a local immune environment. This response not only prevented the establishment of infection in an antigen-independent manner but also promoted the priming of *Leishmania*-antigen specific immunity [reviewed by (21)]. Observations such as these further underscore the urgent need for more basic research to support the rational development of saliva antigen-based vaccines against vector-borne pathogens.

In conclusion, salivary antigens from blood-feeding arthropods that transmit vector-borne diseases have enormous potential as vaccine candidates, alone; in combination with pathogen antigens as was proposed for leishmaniasis (24) or Lyme disease (25); or in combination with vaccines that target endogenous vector antigens (e.g., the conserved protein subolesin (26), or the gut antigen Bm86 already used in the Australian veterinary tick vaccine TickGARD for two decades) and reduce the vector’s viability, fertility, or competence to transmit pathogens. Undoubtedly, combining antigens, either multiple vector-derived antigens, or antigens from pathogens and vectors will lead to more complicated and thus more expensive vaccines. However, the additive or potentially synergistic effect that may be created by targeting vector-borne pathogens at multiple levels carries the potential of finally having a significant impact on some of the most burdensome vector-borne diseases.

## Disclaimer

Views expressed are not to be construed as official policy of the National Institutes of Health (NIH).
